# Influencers of Achievement of Remission and Low Disease Activity in Response to Disease-Modifying Antirheumatic Drugs (DMARDs) in Patients With Rheumatoid Arthritis

**DOI:** 10.7759/cureus.71955

**Published:** 2024-10-20

**Authors:** George O Oyoo, Anastasia Guantai, Robert J Moots, Faith Okalebo, George O Osanjo

**Affiliations:** 1 Department of Clinical Medicine and Therapeutics, University of Nairobi, Nairobi, KEN; 2 Department of Pharmacology, Clinical Pharmacy and Pharmacy Practice, University of Nairobi, Nairobi, KEN; 3 Rheumatology, Edge Hill University, Liverpool, GBR

**Keywords:** disease activity score, health assessment questionnaire, low disease activity, remission, rheumatoid arthritis

## Abstract

Background: In this prospective cohort study the objective was to identify the socio-demographic and clinical factors that influence treatment response to disease-modifying antirheumatic drugs (DMARDs) at ambulatory multicenter rheumatology outpatient clinics. The subjects were patients with rheumatoid arthritis satisfying the American College of Rheumatology/European Alliance of Associations for Rheumatology criteria with informed consent.

Materials and methods: Pre-coded data sheets were used to capture socio-demographic and clinical characteristics. Baseline data was collected at time of patient recruitment. Only patients who had complete data at three-month follow-up were included in the study analysis. The study’s outcome was achievement of remission or low disease activity. The study used the adherence in chronic disease scale and European Task Force for Patient Evaluation of General Practice tools to evaluate patient adherence and assessments of health care received.

Data analysis was carried out using Prism 7 and SPSS. Categorical data were regulated as percentages, while continuous data were regulated as means and standard deviation. Prevalence (at 95% CIs) of various socio-demographic and clinical characteristics were calculated comparisons of socio-demographic characteristics, clinical characteristics between patients into achieved primary/secondary outcomes and those who didn’t were carried out using the chi-square statistic (for categorical variables) and independent student T-test (for continuous variables). Logistic regression was performed to estimate the impact of moderator variables on study outcomes and to calculate adjusted odds ratio (OR) with corresponding 95% CI. Throughout analysis α < 0.05 was considered statistically significant.

Results: A total of 206 patients were included. The mean age was 51.2 ± 15.1 years; mostly females (n=188 patients, 91.3%). Majority had attained post-primary education (n=172 patients, 83.5%). Only 74 patients (35.9%) had formal professional employment, while only six patients (3%) paid for healthcare via government-funded/private insurance.

At recruitment, nearly half of the included patients had moderate to severe disability. Majority of patients had elevated baseline erythrocyte sedimentation rate (ESR) and C-reactive protein (CRP). Most of the patients (90.3%) had a positive rheumatoid factor test at recruitment, while 58% had a positive anti-cyclic citrullinated peptide test. Majority had moderate disease activity. Thirty-three patients were in remission, 9.7% had low disease activity while 12.6% had high disease activity. Majority of patients (94.2%) were on non-steroidal anti-inflammatory drugs, non-biological DMARDs (80.6%) and systemic corticosteroids (54.9%). Mean duration of follow-up was 4.6 months. At follow-up, 37.9% found the system to be acceptable, 63.6% found the system to be problematic. Majority of the patients reported to have been adherent to therapy (high adherence: 7.8%; moderate adherence: 86.9%). The proportion of patients who achieved remission or low disease activity increased significantly at three-month follow-up.

Conclusion: Having shorter disease duration, lower unemployment rates, higher income, lower non-adherence rates, having a positive outlook towards the healthcare system, normal CRP baseline, normal ESR baseline, and lower baseline of functional disability were significantly associated with increased chances of low disease activity/remission.

## Introduction

Rheumatoid arthritis (RA) is a chronic autoimmune inflammatory disease characterized by persistent synovitis and joint destruction. RA treatment prevents joint deformities, impaired function and disability [1,2]. Treatment is based on disease-modifying antirheumatic drugs (DMARDs) to reduce disease activity and to obtain disease remission. Conventional synthetic disease-modifying anti-rheumatic drugs (csDMARDs), such as methotrexate (MTX), are first-line drugs usually prescribed for the treatment of RA [3,4]. Three classes of biological DMARDs (bDMARDs) are also used in patients with inadequate response or intolerance to csDMARDs. Both csDMARDs and bDMARDs show clinical effectiveness with acceptable safety in a substantial proportion of RA patients [2]. However, agents with novel mechanisms of action are always in demand, as a consistent proportion of patients are refractory or intolerant to existing agents.

Patients who do not respond to DMARDs are eligible for biologic or targeted therapies, the most commonly prescribed being tumor necrosis factor α inhibitors (anti‐TNF) [5]. Unfortunately, not only does each drug show a significant nonresponse rate in patients, but failure of the treatment may also lead to irreversible joint damage due to uncontrolled inflammation [6]. Therefore, it would be of great benefit to be able to predict treatment response, so that patients can be assigned the right treatment at an early stage.

## Materials and methods

Study setting

This study was undertaken at rheumatology clinics of four centers: Kenyatta National Teaching and Referral Hospital (KNH) and Mater Hospital (MH) in Nairobi; Mombasa Hospital (MsaH) in Mombasa and Aga Khan University Hospital (AKUH) in Kisumu. Kenyatta National Teaching and Referral Hospital (KNH) was the main site of the study. It is a 2,200-bed capacity hospital located in the capital city of Nairobi in Kenya. It is among the biggest teaching and referral hospitals in the Eastern Africa region and the largest in Kenya. It serves as the primary public hospital serving residents of Nairobi and referral patients from different places across Kenya. KNH also serves as the main educational facility for students in the Faculty of Health Sciences, University of Nairobi (UON). The Rheumatology Clinic is one of the specialized clinics in the Department of Medicine at KNH serving clientele referred from all over Kenya. The clinic runs twice a week on Tuesday and Thursday afternoons. Approximately 30 to 50 patients with various rheumatic diseases are seen at the clinic on each clinic day with rheumatoid arthritis comprising an average of five to 10 patients per clinic day. The other three hospitals (MH, MsaH and AKUH-Kisumu) have relatively smaller rheumatology clinics and were included to augment the main site and to enhance patient’s experiences and different characteristics as well as increase the sampling frame.

Study design and duration

This was a multi-center prospective cohort study conducted between June 2021 to December 2022

Study population and selection criteria

The study population included patients presenting to the Rheumatology Clinic of the four study sites, and with a definite diagnosis of RA based on American College of Rheumatology (ACR) or ACR/European Alliance of Associations for Rheumatology (EULAR) criteria (score ≥6).

Inclusion Criteria

This study included patients with the following: Definite diagnosis of rheumatoid arthritis as per ACR or ACR/EULAR criteria (score ≥6); Aged 18 years and above; On any of following treatment modalities: non-steroidal analgesics (NSAIDS), non-biologic disease-modifying anti-rheumatic drugs (NDMARDs), and bDMARDs or corticosteroids; Completed at least three months of treatment and follow-up; Consented to participate in the study

Exclusion Criteria

Patients who declined to consent to participate in the study and those too ill to participate in the study at the time of data collection were excluded.

Ethical considerations

Ethical consideration was an important aspect of the study and received full attention of the researchers. The study was implemented after ethical clearance was obtained from the KNH-UON Ethics and Research Committee (approval P674/12/2020) and also from the administration of the other three study sites (MH, MsaH and AKUH-Kisumu). Participants were informed of their voluntary participation. Each participant agreed to sign an informed consent sheet before commencement of study. This form included a brief overview of the study and the researcher’s contact information for further questions or clarifications. Respondents in the study were informed of their privacy and confidentiality throughout the study. Anonymity was maintained especially in the data presentation by coding of information instead of using patients’ identities. The data was securely stored in a password-protected computer folder. The investigator honoured exclusive rights and charters, in addition to all forms of intellectual property. Additionally, the lead investigator did not accept the use of unverified data, methods, or results without prior authorization and cited all sources of information to avoid plagiarism.

Data collection

Pre-coded data sheets (questionnaire) were used to capture patient information. The questions were written according to Sekaran for which respondents recorded their answers within strictly defined alternatives. The questionnaires contained both structured and unstructured questions designed to obtain valuable information about the patients. Every element in the questionnaire was adapted to address a precise objective, a research question or a hypothetical estimate of the required knowledge. Questionnaires were administered through face-to-face interviews by a trained medical officer. The same medical officer collected data in all the study sites. Adequate measures were put in place that mitigated the spread of coronavirus disease 2019 (COVID-19) infection by using appropriate clothing and maintaining a recommended social distancing in the community. The researcher ensured that a friendly atmosphere of trust and confidence was created to enable the respondents to discuss freely.

Sociodemographic characteristics

The following sociodemographic characteristics were age (in years), sex (male or female), age groups (<40, 40-49, 50-59, ≥60 years), county of residence (Nairobi, Mombasa, Kisumu), level of education (none, primary level, secondary level, tertiary level), housing (renting or owning), mode of payment for healthcare services (government funding, private insurance, self-pay or family support), occupation (professional employment, businessperson, students, unemployed), level of income (<5000, 5000-19999, 20000-49999, 50000-99999, 100000-149999, >150000 Kenyan shillings) and smoking status.

Clinical characteristics

Data captured included the duration of disease (diagnosis to enrollment into the study), level of functional disability, erythrocyte sedimentation rate (ESR) level, C-reactive protein (CRP) levels, retroviral disease status, rheumatoid factor (RF) positivity, anti-cyclic citrullinated peptide (anti-CCP) positivity, disease activity based on Disease Activity Score-28 (DAS28 score) and treatment modality (non-biological DMARDS ((MTX), hydroxychloroquine (HCQ), leflunomide (LEF), sulfasalazine (SSZ)), biologic DMARDS (adalimumab, etanercept, infliximab, tocilizumab, janus kinase inhibitors (JAKis) and rituximab) and glucocorticoids (oral, intravenous, intramuscular and intra-articular)).

The level of functional disability was determined using the Health Assessment Questionnaire Disability Index (HAQ-DI). It is a self-assessment tool that measures functional ability in eight different areas: rising, dressing and grooming, hygiene, eating, walking, reach, grip, and activities of independent living. Each domain is scored from 1 to 5, with a higher score indicating greater degree of functional disability. Levels of ESR and CRP were categorized as either normal or elevated based on pre-defined cut-off points (25 mm/hr for ESR and 10mg/dl for CRP). 

Disease activity was calculated based on the DAS28 score [7]. This was calculated as follows: DAS28=0.56*√(TJC28) + 0.28*√(SJC28) + 0.36*Ln(CRP*10+1) + 0.014*Patient Global, where: TJC28- Tender joint count; SJC28- Swollen joint count; Ln- Log; Patient Global- patient’s global assessment of disease activity.

The final score was categorized into four groups: a) remission: <2.6; b) low disease activity (LDA): ≥2.6 to ≤3.2; c) moderate disease activity (MDA): >3.2 to ≤5.1; d) high disease activity (HDA): >5.1.

Healthcare system-associated factors

The internationally-validated European Task Force for Patient Evaluation of General Practice (EUROPEP) tool was used to evaluate patient assessments of health care received (the clinical performance of the physician and the organization of practice). The questionnaire contains 23 questions covering four domains: medical care, physician-patient relationship, coordination of care and accessibility. Each question is scored from 1 (poor) to 5 (excellent), with a higher score indicating a more acceptable quality of care in that domain. The scores were then converted into percentages and graded as follows: a) 90-100%: Good; b) 80-89%: Acceptable; c) <80%: Problematic.

The robustness (internal consistency reliability) of the EUROPEP tool was assessed using the Cronbach alpha statistic, with a value of >0.80 indicating high degree of reliability.

Adherence

Adherence to treatment was assessed using the Adherence in Chronic Diseases Scale (ACDS) tool, which has been widely validated for use in adult patients on treatment for chronic diseases. The tool contains seven questions: 1-5 are based on patient characteristics to treatment-taking behavior; questions 6 and 7 show the doctor-patient connection that impairs adherence. Each question is scored on a scale of 0 (never) to 4 (always) points. A total score of greater than 26 points indicates high adherence to treatment, while scores 21-26 and <21 points are moderate and low respectively.

## Results

In the first step of our study we analyzed the association between the sociodemographic factors and clinical parameters, heath care related factors and the response to DMARD therapy in RA patients.

Socio-demographic characteristics

The mean age of included patients was 51.2 ± 15.1 years, with majority of patients (n=159 patients, 78%) being above 40 years. Most of them were females (n=188 patients, 91.3%). The mean age at the time of diagnosis of rheumatoid arthritis was 43.3 ± 14.4 years. The interval between time of diagnosis of RA and recruitment into the study (duration of illness) was 7.4 ± 8.6 years (Table 1). Majority of the patients were from Nairobi County (n=187, 90.8%), and had attained post-primary education (n=172 patients, 83.5%). Only 74 patients (35.9%) had formal professional employment. Majority of patients (n=137 patients, 66.5%) were living in rented houses. Only six patients (3%) paid for healthcare via government-funded/private insurance. Majority paid for services out of pocket (n=196 patients, 95.1%). Approximately half of the included patients had a monthly income of >50,000 Kenyan shillings. Only eight patients (3.9%) had history of cigarette smoking (Table 2).

**Table 1:** Age Distribution and County of Residence RA: Rheumatoid arthritis

Characteristic	Total	
Age (years; mean, SD)	At diagnosis of RA	43.3 ±14.4
	At enrollment in the study	51.2±15.1
Age groups (n, %)	<40 years	47 (22.8%)
	40-49 years	46 (22.3%)
	50-59 years	43 (20.9%)
	≥60 years	70 (34%)
County (n, %)	Nairobi	187 (90.8%)
	Mombasa	9 (4.4%)
	Kisumu	10 (4.9%)

**Table 2:** Level of Education and Socioeconomic characteristics of study participants

Characteristics	Total	
Education level (n, %)	None	8 (3.9%)
	Primary level	26 (12.6%)
	Secondary level	49 (23.8%)
	Tertiary level	123 (59.7%)
Housing (n, %)	Renting	137 (666.5%)
	Owning	65 (31.6%)
	Living without paying	4 (1.9%)
Payment for healthcare (n, %)	Government funding	3 (1.5%)
	Private Insurance	3 (1.55)
	Self-pay	196 (95.1%)
	Family support	4 (1.9%)
Occupation (n, %)	Unemployed	19 (19.2%)
	Student	6 (2.9%)
	Housewife	47 (22.8%)
	Business person	43 (20.9%)
	Farmer	17 (8.3%)
	Professional Employment	74 (35.9%)
Income (n, %)	< 5,000	71 (34.5%)
	5,000-19,999	15 (7.3%)
	20,000-49,999	21 (10.2%)
	50,000-99,999	65 (31.6%)
	100,000-149,999	27 (13.1%)
	>150,000	7 (3.4%)
Smoking (n, %)	Yes	8 (3.9%)
	No	198 (96.1%)

Clinical characteristics

The mean HAQ-DI was 2.5±0.9. Nearly half of the included patients (n=98 patients, 47.6%) had an overall HAQ-DI score of more than 2.5, indicating moderate to severe disability (Table 3). Three patients (1.5%) had retroviral disease at enrolment. The mean ESR and CRP levels were 36.5±23.6 and 14.5±24.2 respectively, with majority of patients presenting with elevated levels (n=128, 62.1% and n=151, 73.3% respectively). Most of the patients had a positive rheumatoid factor (RF) test at recruitment (n=186 patients, 90.3%), while only 113 patients (58.5%) had a positive anti-cyclic citrullinated peptide (anti-CCP) test (Table 4). The mean DAS28 score at recruitment was 4.0±1.5. Majority had MDA (n=127 patients, 61.7%). Thirty-three patients (16%) were in remission, 20 (9.7%) had LDA while 26 (12.6%) had HDA.

**Table 3:** Health Assessment Questionnaire Disability Index (HAQ-DI) scores for included patients The higher the score, the greater the disability.

Question	Domain	Score
How did your arthritis affect your ability to carry out your daily life this week?	General	3.48±0.93
Dress yourself, including tying belt, shoelaces, and pyjama and doing buttons?	Dressing	2.21±1.10
Get in and out of bed, stand up from chair?	Arising	2.21±1.07
Lift a full cup or glass to your mouth, cut your meat?	Eating	2.26±1.11
Walk outdoors on flat ground, climb 5 steps?	Walking	1.78±1.00
Wash and dry your entire body?	Hygiene	2.22±1.18
Squat in the toilet or sit cross-legged on the floor?	Reach	2.36±1.15
Bend down to pick up clothing from the floor?	Reach	2.74±1.21
Turn a tap on and off, open a previously opened jar?	Grip	2.28±1.09
Get in and out of car/ matatu?	Daily activity	3.36±1.36
Walk three kilometers?	Daily activity	2.63±1.25
Shop in a vegetable market?	Daily activity	2.36±1.15
Climb a flight of stairs?	Daily activity	2.29±1.12

**Table 4:** Clinical characteristics of study participants ESR: erythrocyte sedimentation rate, CRP: C-reactive protein, anti-CCP: anti-cyclic citrullinated peptide, DMARDs: disease-modifying antirheumatic drugs, DAS28: Disease Activity Score-28, NSAIDs: nonsteroidal anti-inflammatory drugs

Characteristic		Total
Retroviral disease (n, %)	Yes	3 (1.5%)
	No	203 (98.5%)
ESR (n, %)	Elevated	128 (62.1%)
	Normal	78 (37.9%)
CRP (n, %)	Elevated	151 (73.3%)
	Normal	55 (26.7%)
Rheumatoid factor (n, %)	Positive	186 (90.3%)
	Negative	20 (9.7%)
Anti-CCP (n, %)	Positive	113 (58.5%)
	Negative	80 (41.5%)
Disease activity (n, %)	Remission (DAS28 score <2.6)	33 (16%)
	Low disease activity (DAS28 score 2.6-3.2)	20 (9.7%)
	Moderate disease activity (DAS28 score 3.3-5.1)	127 (61.7%)
	High disease activity (DAS28 score >5.1)	26 (12.6%)
Drugs	NSAIDs	194 (94.2%)
	Steroids	113 (54.9%)
	Non-biological DMARDs	166 (80.6%)
	Biological DMARDs	24 (11.7%)
Biological DMARDs	Tocilizumab	15 (7.3%)
	Infliximab	3 (1.5%)
	Rituximab	4 (1.9%)
	Golimumab	2 (1%)
	Adalimumab	2 (1%)
	Ertanecept	1 (0.5%)
Non-biological DMARDs	Leflunamide	99 (48.1%)
	Hydroxychloroquine	95 (46.1%)
	Methotrexate	75 (36.4%)
	Sulphasalazine	25 (12.1%)

Majority of patients were on NSAIDs (n=194 patients, 94.2%), non-biological DMARDs (n=166 patients, 80.6%) and systemic corticosteroids (n=113 patients, 54.9%). Twenty-four patients (11.7%) were on biological DMARDs. The commonest non-biological DMARD was leflunamide (n=99 patients, 48.1%), followed by hydroxychloroquine (n=95 patients, 46.1%), methotrexate (n=75 patients, 36.4%) and sulphasalazine (25 patients, 12.1%). The most commonly used biological agent was tocilizumab (n=15 patients, 7.3%) (Table 4).

Clinical characteristics at follow-up

**Figure 1:**
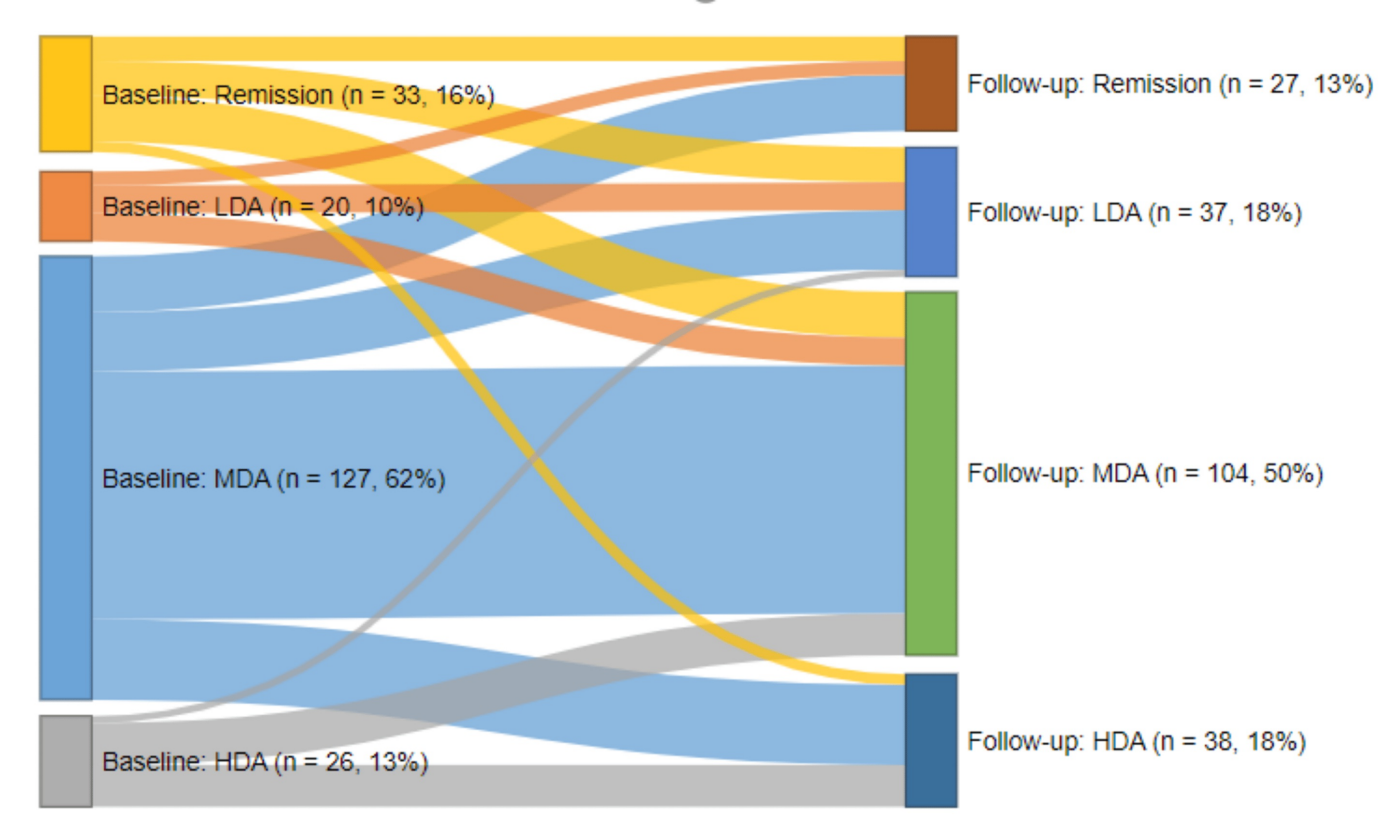
Trajectory of disease severity for included patients LDA: low disease activity, MDA: moderate disease activity, HAD: high disease activity

The mean duration of follow-up was 4.6 months (140±61 days). Compared with baseline, there was a significant reduction in the proportion of patients with elevated ESR (62.1% vs 56.8%, p<0.001), elevated CRP (73.3% vs 64.5%, p=0.002) and moderate/severe disability (47.6% vs 42.2%, p<0.001) at follow-up. There was a slight reduction in the mean DAS28 score at follow-up (4.0±1.5 vs 3.9±1.2, p=0.502) (Table 5). The proportion of patients who achieved primary outcome (remission or low disease activity) increased significantly at three-month follow-up (25.6% vs 31%, p<0.001) (Table 5). Nearly half of included patients (n=98 patients, 47.6%) remained in same disease severity category at follow-up. Fifty-one patients (24.8%) demonstrated improvement by shifting to a less severe disease category, while 57 (27.7%) deteriorated (had a more severe disease category at follow-up) (Figure 1).

**Table 5:** Clinical characteristics of patients at follow-up ESR: Erythrocyte sedimentation rate, CRP: C reactive protein, LDA: low disease activity, MDA: moderate disease activity, HAD: high disease activity, DAS28: Disease Activity Score-28, HAQ-DI: Health Assessment Questionnaire Disability Index

Variable		Baseline	Follow-up	P-value
ESR	Normal	78 (37.9%)	89 (43.2%)	p<0.001
	Elevated	128 (62.1%)	117 (56.8%)	
CRP	Normal	55 (26.7%)	129 (64.5%)	p=0.002
	Elevated	151 (73.3%)	71 (35.5%)	
HAQ-DI Score	No disability	108 (52.4%)	119 (57.8%)	p<0.001
	Disability	98 (47.6%)	87 (42.2%)	
DAS-28 severity	Remission/LDA	53 (25.7%)	64 (31.1%)	p<0.001
	MDA/HDA	153 (74.3%)	142 (68.9%)	

Patients’ assessment of health system at follow-up

Overall, a total of 75 patients (37.9%) found the system to be acceptable. Majority (n=131 patients, 63.6%) found the system to be problematic. Of the 22 items, only nine (40.9%) had a score of >80% representing acceptability, while the rest were identified as problematic. Domain 2 (physician-patient relation) was found to be most acceptable by patients with five out of seven questions having a score of >80%. This was followed by domain 3 (coordination of care, one out of two questions), domain 4 (accessibility, one out of four). Domain 1 (medical care) scored the least (only two out of nine questions with score >80%) (Table 6). The overall Cronbach alpha value was 0.88, indicating a high degree of reliability (“internal consistency”) of the EUROPEP tool in the current study (Table 6).

**Table 6:** Patients’ assessment of health system using European Task Force for Patient Evaluation of General Practice (EUROPEP) questionnaire Domains: 1- Medical care; 2- physician-patient relationship; 3- coordination of care; 4- accessibilty. The higher the score, the more acceptable the quality of care of that domain.

Domain	Question	Mean	SD	Score	Cronbachs α
1	Q4: Involving you in decisions about your medical care	3.9	0.68	78.4	0.88
1	Q7: Quick relief of your symptoms	3.4	0.53	67.7	0.88
1	Q8: Helping you to feel well so that you can perform your normal daily activities	3.4	0.53	68.1	0.87
1	Q9: Thoroughness	4.5	0.54	88.7	0.87
1	Q11: Offering you services for preventing diseases	4	0.53	80.6	0.88
1	Q12: Explaining the purpose of tests and treatments	3.7	0.8	72.8	0.87
1	Q13: Telling you what you wanted to know about your symptoms and/or illness	3.7	0.76	73.2	0.87
1	Q15: Helping you understand the importance of following his or her advice	3.5	0.94	70.5	0.88
1	Q16: Knowing what she/he had done or told you during previous contacts	3.7	0.75	74.4	0.87
2	Q1: Making you feel you had time during consultation	4.7	0.54	92.6	0.88
2	Q2: Interest in your personal situation	4.6	0.54	91.9	0.87
2	Q3: Making it easy for you to tell him or her about your problems	4.2	0.58	82.6	0.88
2	Q5: Listening to you	4.2	0.55	84.4	0.88
2	Q6: Keeping your records and data confidential	4	0.52	79.9	0.88
2	Q10: Physical examination of you	4.5	0.52	88.7	0.87
2	Q14: Help in dealing with emotional problems related to your health status	3.4	0.69	68.4	0.87
3	Q17: Preparing you for what to expect from specialist or hospital care	4	0.49	79.2	0.88
3	Q18: The helpfulness of staff (other than the doctor)	4.1	0.57	81.5	0.88
4	Q19: Getting an appointment to suit you	3.9	0.59	77.2	0.88
4	Q20: Getting through to the practice on the phone	2.4	0.77	48.3	0.89
4	Q21: Being able to speak to the GP on the telephone	2.4	0.56	48.6	0.89
4	Q22: Waiting time in the waiting room	4	0.37	80	0.89

Adherence

**Figure 2:**
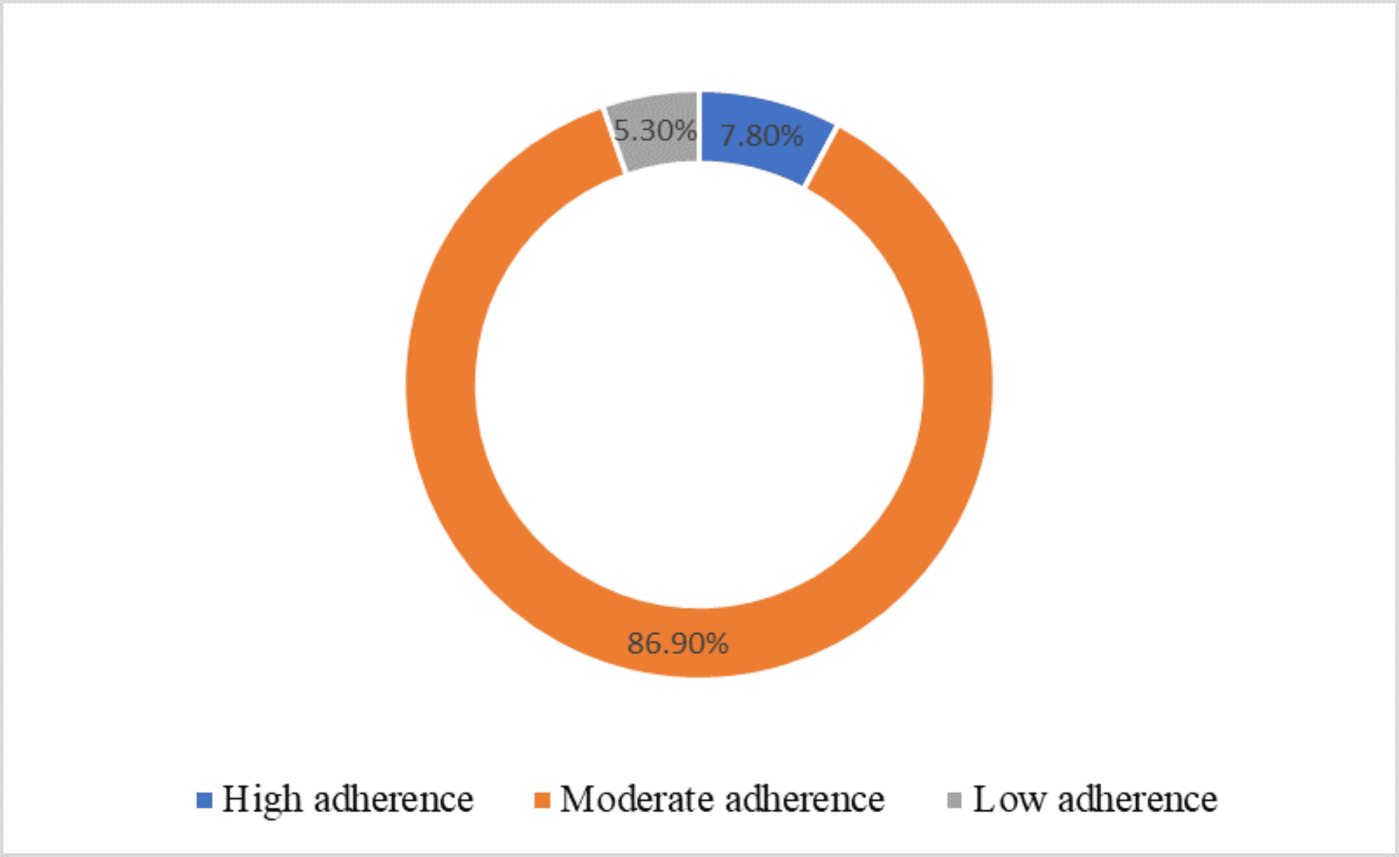
Doughnut Presentation of Adherence

Majority of the patients reported to have been adherent to therapy (high adherence: n=16 patients, 7.8%; moderate adherence: n=179 patients, 86.9%). A total of 11 patients (5.3%) reported non-adherence to therapy (Figure 2).

Comparison of socio-demographic characteristics

Patients who achieved primary outcome (remission/low disease activity) had significantly lower unemployment rate (25.8% vs 74.2%, p=0.041) and higher income (proportion of patients earning ≥20,000 Ksh: 73.4% vs 51.4%, p=0.004) than those with moderate-high disease activity (Table 7). The age at diagnosis (42.0±13.4 vs 43.9±14.8, p=0.398), level of education (none/primary: 7.8% vs 20.4%; post-primary: 92.2% vs 79.6%, p=0.066), mode of payment of healthcare services (government/private insurance: 3.1% vs 2.8%; self-pay: 96.9% vs 97.2%, p=0.605) and cigarette smoking (3.1% vs 4.2%, p=0.524) did not differ significantly between the two groups (Table 7).

**Table 7:** Comparison of socio-demographic characteristics between patients with favorable outcomes (remission/low disease activity) vs unfavorable outcome (moderate-high disease activity) at follow-up LDA: low disease activity, MDA: moderate disease activity, HAD: high disease activity, SD: standard deviation, Ksh: Kenyan Shillings, #: Student's t-test, £: Chi Square statistic

Characteristic		Remission/LDA (n=64 patients)	MDA/HDA (n=142 patients)	P-value
Age at diagnosis (years) (mean, SD)		42.0±13.4	43.9±14.8	p=0.398^# ^
County	Nairobi	57 (89.1%)	130 (91.5%)	p=0.381^£ ^
	Mombasa	2 (3.1%)	7 (4.9%)	
	Kisumu	5 (7.8%)	5 (3.5%)	
Education	None/primary	5 (7.8%)	29 (20.4%)	p=0.066^£^
	Post-primary	59 (92.2%)	113 (79.6%)	
Healthcare payment	Government/private insurance	2 (3.1%)	4 (2.8%)	p=0.605^£ ^
	Self-pay	62 (96.9%)	138 (97.2%)	
Income	<20,000 Ksh.	17 (26.6%)	69 (48.6%)	p=0.004^£^
	≥20,000 Ksh.	47 (73.4%)	73 (51.4%)	
Occupation	Unemployed/student/housewife/self-employed	34 (25.8%)	98 (74.2%)	p=0.041^£^
	Professional employment	30 (40.5%)	44 (59.5%)	
Smoking	Yes	2 (3.1%)	6 (4.2%)	p=0.524^£^
	No	62 (96.9%)	136 (95.8%)	

Clinical characteristics and healthcare system-associated factors

Patients who achieved primary outcome (remission/low disease activity) had significantly shorter duration of disease (4.8±4.5 vs 8.5±9.7 years, p=0.004), were more likely to have normal baseline ESR (56.3% vs 29.6%, p<0.001) and CRP (35.9% vs 22.5%, p<0.001) at diagnosis, had a lower baseline disease activity score (3.31±0.98 vs 4.26±1.55, p<0.001) and lower baseline degree of functional disability (1.98±0.74 vs 2.69±0.87, p<0.001) than those with moderate-high disease activity (Table 8). They also had lower rates of non-adherence to treatment (0.0% vs 17.7%, p=0.015), and had a more positive outlook (good/acceptable) of the healthcare system based on EUROPEP score (50.0% vs 30.3%, p=0.023). No significant differences in rheumatoid factor positivity (93.8% vs 88.7%, p=0.233), anti-CCP positivity (59.7% vs 58.0%, p=0.876) and drug regimen (biologic DMARDS: 9.4% vs 12.7%, p=0.334; non-biologic DMARDS: 78.1% vs 81.7%, p=0.337; NSAIDs: 93.4% vs 94.4%, p=0.543; steroids: 56.3% vs 54.2%, p=0.880) were noted between the two groups (Table 8).

**Table 8:** Comparison of clinical characteristics, adherence and health system evaluation between patients with favorable outcomes (remission/low disease activity) vs unfavorable outcome (moderate-high disease activity) at follow-up ESR: erythrocyte sedimentation rate, CRP: C reactive protein, LDA: low disease activity, MDA: moderate disease activity, HAD: high disease activity, DAS28: Disease Activity Score-28, HAQ-DI: Health Assessment Questionnaire Disability Index, DMARDs: disease-modifying antirheumatic drugs*, *NSAIDs: nonsteroidal anti-inflammatory drugs*, *anti-CCP: anti-cyclic citrullinated peptide, SD: standard deviation, Ksh: Kenyan Shillings, #: Student's t-test, £: Chi Square statistic, EUROPEP: European Task Force for Patient Evaluation of General Practice

Characteristic		Remission/LDA (n=64 patients)	MDA/HDA (n=142 patients)	p-value
Duration of disease (years) (mean, SD)		4.8±4.5	8.5±9.7	p=0.004^#^
Retroviral disease	Yes	1 (1.6%)	2 (1.4%)	p=0.675^£^
	No	63 (98.4%)	140 (98.6%)	
ESR	Elevated	28 (43.8%)	100 (70.4%)	p<0.001^£^
	Normal	36 (56.3%)	42 (29.6%)	
CRP	Elevated	41 (65.6%)	110 (77.5%)	p=0.044^£^
	Normal	23 (35.9%)	32 (22.5%)	
Rheumatoid factor	Positive	60 (93.8%)	126 (88.7%)	p=0.233^£^
	Negative	4 (6.2%)	16 (11.3%)	
Anti-CCP	Positive	37 (59.7%)	76 (58.0%)	p=0.876^£^
	Negative	25 (40.3%)	55 (41.9%)	
Baseline DAS-28 Score		3.31±0.98	4.26±1.55	p<0.001^#^
Baseline HAQ-DI (disability) Score		1.98±0.74	2.69±0.87	p<0.001^#^
Biologic DMARDS	Yes	6 (9.4%)	18 (12.7%)	p=0.334^£^
	No	58 (90.6%)	124 (87.3%)	
Non-biologic DMARDS	Yes	50 (78.1%)	116 (81.7%)	p=0.337^£ ^
	No	14 (21.9%)	26 (18.3%)	
NSAIDS	Yes	60 (93.4%)	134 (94.4%)	p=0.543^£ ^
	No	4 (6.6%)	8 (5.6%)	
Steroids	Yes	36 (56.3%)	77 (54.2%)	p=0.880^£ ^
	No	28 (43.8%)	65 (45.8%)	
Adherence	Low	0 (0.0%)	11 (17.7%)	p=0.015^£^
	High	64 (100.0%)	131 (92.3%)	
EUROPEP	Good/acceptable	32 (50.0%)	43 (30.3%)	p=0.023^£ ^
	Problematic	32 (50.0%)	99 (69.7%)	

Predictors of remission/low disease activity 

In unadjusted (univariate) regression analysis, having formal employment (OR=1.97, 95% CI 1.07-3.60, p=0.019), higher income (≥20,000 Ksh.) (OR=2.61, 95% CI 1.37-4.98, p=0.019), adherence (OR=2.65, 95% CI 1.12-6.30, p=0.027) and positive outlook towards healthcare system (OR=2.08, 95% CI 1.18-3.66, p=0.011) were associated with increased chances of low disease activity/remission (Table 9). On the other hand, longer disease duration (OR=0.93, 95% CI 0.88-0.98, p=0.004), elevated baseline ESR (OR=0.33, 95% CI 0.18-0.60, p<0.001), elevated baseline CRP (OR=0.52, 95% CI 0.27-0.99, p=0.046), higher baseline functional disability (OR= 0.33, 95% CI 0.22-0.51, p<0.001) and higher DAS28 disease activity score (OR=0.49, 95% CI 0.37-0.67, p<0.001) were associated with lower chances of low disease activity/remission.

**Table 9:** Summary of findings that significantly led to remission/LDA ESR: erythrocyte sedimentation rate, CRP: C reactive protein, LDA: low disease activity, DAS28: Disease Activity Score-28, Ksh: Kenyan Shillings, EUROPEP: European Task Force for Patient Evaluation of General Practice

Characteristics	p-value
Lower unemployment rate	p<0.041
Higher income (≥20,000 Ksh.)	p<0.004
Shorter duration of disease	p<0.004
Normal baseline ESR	p<0.001
Normal baseline CRP	p<0.001
Lower baseline disease activity score (DAS28)	p<0.001
Lower baseline degree of functional disability	p<0.001
Lower rates of non-adherence to treatment	p<0.015
Having positive outlook (good/acceptable) of the healthcare system based on EUROPEP score	p<0.023
Having formal employment	p<0.019

## Discussion

The study analysed a total of 206 patients who met the inclusion criteria with a mean age of (51.2 ± 15.1) years, majority of patients (n=159 patients, 78%) had attained 40 years and above with a female preponderance (n=188 patients, 91.3%). The mean DAS28 score at recruitment was 4.0±1.5. Majority had MDA (n=127 patients, 61.7%), 33 patients (16%) were on remission, 20 (9.7%) had LDA while 26 (12.6%) had HDA. A total of 53 patients on follow-up had remission and LDA from the study. The factors that might have influenced remission from the study are summarized in Table 9.

The study recorded significantly lower rates of non-adherence to treatment (p<0.015) in those patients who had low disease activity/remission. This study established that 86.9% majority of the patients had MDA, 7.8% had HDA while 5.3% poorly adhered to RA therapy. Consequently, the majority of the patients with MDA and HDA experienced better treatment response, thus resulting in LDA/remission. Most studies have shown that adherence to medication in patients with rheumatoid arthritis is low, varying from 30 to 80% [8]. Improving on adherence to therapy could therefore dramatically improve the efficacy of drug therapy [8,9]. Adherence to medication therapy is important to reach the desired treatment outcome and for the management of RA, especially at the start of treatment [10]. Likewise, Arshad et al. have indicated that higher medication adherence with RA exhibits achievement of lower disease activity during treatment with DMARDs [9]. The most desirable target measure of disease activity is remission, which signifies a condition of negligible or no inflammatory activity, absolute arrest of structural joint damage and the optimum achievable reversal of disability [11]. This study had a remission of 16% while Larid et al. study observed an increased remission (53%) with fewer corticosteroids and more biologics therapy in rheumatoid arthritis in a seven year follow-up in real-life conditions at the Poitiers University Hospital [12].

Another contributing factor to better treatment outcomes and remission is favorable social determinants of health (SDOH). A compelling body of public health research suggests that clinical care accounts for approximately 20% of health outcomes, while socioeconomic, behavioral, and environmental factors determine the remaining 80% [13-15]. These SDOH encompass “the circumstances, in which people are born, grow up, live, work, and age” and have a significant impact on the health outcomes of many chronic diseases, including RA [14,16]. This study reveals the interplay of favorable SDOH factors believed to have enhanced remission and better treatment outcomes. The study reveals that those patients with high-income levels (≥20,000 ksh., p<0.004), lower rates of unemployment (p<0.041) and those with formal employment (40.5%) had significant remission/LDA outcomes. Chronic illnesses such RA are constantly impoverishing families across the world and have worse economic implications and ramifications on households. Therefore, populations with higher income and lower rates of unemployment makes individuals capable of acquiring quality healthcare in terms of early attention for diagnostic services and follow-up on treatment.

The majority of patients did not smoke (96.9%) and this was helpful in remission. The study recorded 3.9% of patients who smoke. This result is consistent with areas such as education, social and work conditions, relationship with lifestyle choices (tobacco, food, alcohol consumption) and other factors such as SODH factors which are significant in the development of RA [17,18].

There is also prevailing evidence that healthcare costs may hamper treatment outcomes [18] and thus those patients in high socioeconomic positions (SEPs) are more likely to acquire healthcare unlike those compelled to use out-of-pocket (OOP) expenses. Having a positive outlook (good/acceptable) of the healthcare system based on EUROPEP score was significantly associated with remission (p<0.023). It also follows that this bracket of patients will have better adherence to their medication and also maintain clinic visits and any other additional clinical requirements like lab tests, physiotherapy, chiropractor, etc. [10].

The study also analysed clinical factors that contribute to disease progression and their evaluation to pinpoint aspects that contributed to remission. The study established mean DAS28 score at recruitment was (4.0±1.5) with a remission of 16%. There was marked improvement in those who achieved remission/LDA as they significantly experienced shorter duration of disease (p<0.004). Studies have shown that the use of appropriate anti-rheumatic drugs is important, especially in patients with early RA before six months’ time, as this is the 'window of opportunity' that helps modify the disease progression [19]. The study by Ajeganova and Huizinga shows that sustained remission is important in shorter duration of disease and especially in early identification as it offers hope that it can change the course of the disease of rheumatoid arthritis and potentially eradication of rheumatoid arthritis [20].

The study recorded normal baseline of CRP (p<0.001) and normal baseline of ESR (p<0.001) as factors that significantly led to remission. Serum ESR and CRP are considered important biomarkers in determining the diagnosis and treatment of RA. Currently, CRP and ESR are common markers of inflammation used to diagnose inflammation [21]. Both markers change, but CRP is more important because of the rapid changes it makes in response to changes in the severity of inflammation. Importantly, a new method of serum CRP detection using a high-sensitivity CRP kit (hs-CRP) can detect mild disease even in difficult-to-treat asymptomatic patients [21].

Lower baseline disease activity score DAS28 (p<0.001) and lower baseline degree of functional disability (p<0.001) were significant in attaining remission. Treatment with DMARDs offers good clinical response in RA patients and thus subsequent clinical evaluations on CRP and ESR should be minimal. Most studies have shown that treatment of RA with DMARDs aims to reduce systemic inflammation and improve disease activity [22]. As a measure of systemic inflammation, it would be expected that CRP and ESR levels will fall in response to treatment and indeed this is observed during treatment with the different DMARD classes [22,23]. Also, Arshad et al. showed that DMARDs in RA patients exhibit successful disease activity during treatment [9]. The best measure of disease activity is remission, which indicates a state of controlled or inactive inflammatory activity, complete arrest of structural joint damage and the best achievable reversal level of disability [23].

The study also established that having a positive outlook (good/acceptable) of the healthcare system based on EUROPEP (OR=2.08, 95% CI 1.18-3.66, p<0.023) significantly influenced remission/LDA in RA patients. Studies by Grol et al. show overriding evidence that a better healthcare system produces better outcomes in terms of patient’s satisfaction with their treatment [24]. This study used the internationally-validated EUROPEP questionnaire assessing the clinical performance of the physician and the organizational setting of practice. The overall Cronbach alpha value was 0.88, indicating a high degree of reliability (“internal consistency”) of the EUROPEP tool in the current study. Out of the 23 questions assessed four domains: medical care, physician-patient relationship, coordination of care and accessibility. The study registered significant positive outlook (good/acceptable) of the healthcare system based on EUROPEP score. Data shows a total of 121 (58.7%) was problematic, 76 (36.9%) acceptable, and nine (4.4%) good. A total of 84 patients (36.9%) and 4.4% found the system to be acceptable and good respectively showing a more positive outlook on the healthcare system could be construed to have positive outcomes and remission on treatment. Several authors argue that satisfaction and the result in terms of the patient’s health status are related terms [25]. Thus, the present study has shown a total of 41.3% (36.9% and 4.4%) making desirable margins for acceptability and good scores and this could explain the bracket of patients who had end-stage remission on care. In addition, the HAQ-DI is a subjective tool and may be influenced by social, economic and cultural factors [19]. It is known that sociodemographic, environmental and health conditions may influence physical function of RA patients [14].

## Conclusions

Achieving and maintaining remission in RA patients is likely to be associated with substantial economic benefits. This study has shown that those who had moderate to high adherence to medication, shorter disease duration, normal baseline CRP and ESR, lower disease activity and lower degree of functional disability had better remission. Similarly, a positive outlook of the healthcare system, better satisfaction with healthcare system and higher SEPs increase the chances of achieving remission.
